# Influence of Water Availability and *Bacillus* Inoculation on Morphophysiology, Biochemistry and Secondary Metabolite in *Lippia origanoides* Kunth

**DOI:** 10.1111/ppl.71132

**Published:** 2026-09-24

**Authors:** Henarmmany Cristina Alves de Oliveira, Larisse Bianca Soares Pereira, Manuela Neri, Igor Alexsander de Melo Pimentel, Claudio Augusto Gomes da Câmara, Marcilio Martins de Moraes, Flávia Carolina Lins da Silva, Elineide Barbosa de Souza, Luiz Palhares Neto, Cláudia Ulisses

**Affiliations:** ^1^ Department of Biology Federal Rural University of Pernambuco (UFRPE) Recife Pernambuco Brazil; ^2^ National Institute of Biotechnology for the Mineral Sector (INABIM) Recife Pernambuco Brazil; ^3^ Department of Agronomy Federal Rural University of Pernambuco (UFRPE) Recife Pernambuco Brazil; ^4^ Department of Chemistry Federal Rural University of Pernambuco (UFRPE) Recife Pernambuco Brazil; ^5^ Department of Biological Sciences Southwest Bahia State University (UESB) Jequié Bahia Brazil

**Keywords:** co‐cultivation, essential oil (EO), osmoprotective compounds, reactive oxygen species (ROS), terpenoids

## Abstract

*Lippia origanoides*, commonly known as ‘alecrim‐pimenta’, is an aromatic shrub with pharmacological and agrochemical potential. Bacterial treatments enhance the overall stress response of the plant, affecting morphophysiological and biochemical parameters and stimulating the biosynthesis of secondary metabolites of industrial relevance. This study aimed to determine the influence of bacterial inoculation on the morphophysiology, biochemistry, yield, and composition of *L*. *origanoides* essential oil under varying water availability. Plants were inoculated with different *Bacillus* strains: *B. cereus* (C240), *B. megaterium* (RAB2), *B. subtilis* (IN‐937b), and a consortium of these bacterial strains. At 33 days after inoculation, plants were irrigated to 25 or 75% of pot capacity for 37 days, resulting in a total experimental period of 70 days. Biometric evaluations were performed at 16, 40, 54, and 68 days, physiological assessments at 19, 41, 55, and 69 days, and biochemical and essential oil analyses at the end of the experiment. Data were analyzed using two‐way analysis of variance with Tukey and Bonferroni tests (*p* ≤ 0.05). Water availability was the main factor affecting morphophysiological parameters, as well‐irrigated plants produced more vegetative and reproductive organs and water‐limited plants accumulated osmoprotective compounds. Although the essential oil yield did not differ significantly among treatments, plants inoculated with bacterial strains produced terpenoids with antioxidant and insecticidal properties. The results demonstrate the potential of *Bacillus* inoculants to enhance secondary metabolism in *L*. *origanoides*, supporting sustainable bioproduct development and biotechnological applications.

## 1 Introduction

Plants are exposed to environmental conditions that induce stress during growth and development, with water restriction being one of the most significant. Reduced water availability affects the overall plant metabolism and leads to oxidative imbalance due to the increased production of reactive oxygen species (ROS), such as hydrogen peroxide (H_2_O_2_) (Wang et al. 2024). However, plants have developed morphological, physiological, and biochemical mechanisms to mitigate the deleterious effects of water deficit (Rehman et al. 2024; Sandeep and Godi 2023; Wang et al. 2024). The physiological strategies that plants employ include adjustments in gas exchange responses, such as the modulation of stomatal conductance (*g*_s_) dynamics to reduce leaf transpiration. This decreases the photosynthetic rate and growth, which reduces leaf production (Bisht et al. 2022; Tiepo et al. 2018).

Biochemically, stress tolerance responses include the accumulation of osmoregulatory molecules, such as soluble carbohydrates and amino acids, including proline (Gao et al. 2020), and the production of cell signaling molecules. These responses also include the enhancement of antioxidant system activity, which is achieved through the positive regulation of antioxidant enzymes, such as superoxide dismutase (SOD), catalase (CAT), and ascorbate peroxidase (APX) (Dodd et al. 2010; Rukh et al. 2025). The enhancement of the plant antioxidant system prevents potential oxidative damage, as evidenced by the low malondialdehyde (MDA) content in plant tissues, an important plant stress biomarker (Abdelaal et al. 2021; Heath and Packer 1968).

The water deficit tolerance of plants can be influenced by associations with bacterial inoculants, which may enhance plant performance and confer synergistic benefits under stress conditions (Rukh et al. 2025). Relevant bacterial genera include *Bacillus*, *Arthrobacter*, *Streptomyces*, *Azospirillum*, *Paenibacillus*, and *Pseudomonas* (Numan et al. 2018). Co‐cultivation with these bacterial inoculants positively influences physiological (Bisht et al. 2022), morphological (Tiepo et al. 2024), and biochemical parameters (Gagné‐Bourque et al. 2016; Tiepo et al. 2018) and affects essential oil (EO) production in plants with medicinal and agrochemical potential (Araújo et al. 2020; Çakmakçı et al. 2020). Defense responses activated under stress influence the quality and quantity of the bioactive compounds present in the EO, significantly altering the secondary metabolite (SM) composition (Kumar et al. 2022).

*Lippia* (Verbenaceae) is one of several genera of EO producing plants, encompassing approximately 200 shrub and herbaceous species found in tropical and subtropical regions (Soares et al. 2017). *Lippia origanoides* Kunth, known as “alecrim‐pimenta,” is a shrub with aromatic leaves native to Northeastern Brazil. This species is recognised for its antibacterial (Ribeiro et al. 2021), antifungal (Brandão et al. 2021), and agrochemical potential (Camilo et al. 2022; Cavalcanti et al. 2010). The potential of *L*. *origanoides* is primarily due to the biosynthesis of the major components of its EO and the production of volatile SMs. These compounds are typically terpenoids derived from the methylerythritol phosphate (MEP) and mevalonate (MVA) biosynthetic pathways (Semenzato and Fani 2024). Terpenoids are the most abundant SMs in the genus *Lippia* (Melo et al. 2022), which is attributed to the formation of compounds that confer medicinal and agrochemical properties, such as thymol, carvacrol, and β‐caryophyllene. Enhancing the plant stress tolerance and physiological capacity may represent studies for obtaining a higher quality and greater yield of SMs extracted from plants (Mahajan et al. 2020).

In this context, the present study aimed to determine the effects of bacterial inoculation, both individually and in combination, on the morphophysiological and biochemical responses and the production and quality of the EO of *L*. *origanoides* subjected to water restriction. To this end, the following hypotheses were formulated: (1) bacterial inoculation enhances the morphophysiological performance of *L*. *origanoides* under water stress by improving growth and stomatal regulation; (2) under water restriction, bacterial inoculation modulates plant stress responses by increasing the antioxidant activity and osmolyte accumulation, reducing stress‐induced damage; and (3) the interaction between water stress and bacterial inoculation alters EO production and chemical composition, affecting yield and the concentration of compounds associated with stress responses.

## 2 Materials and Method

**FIGURE 1:**
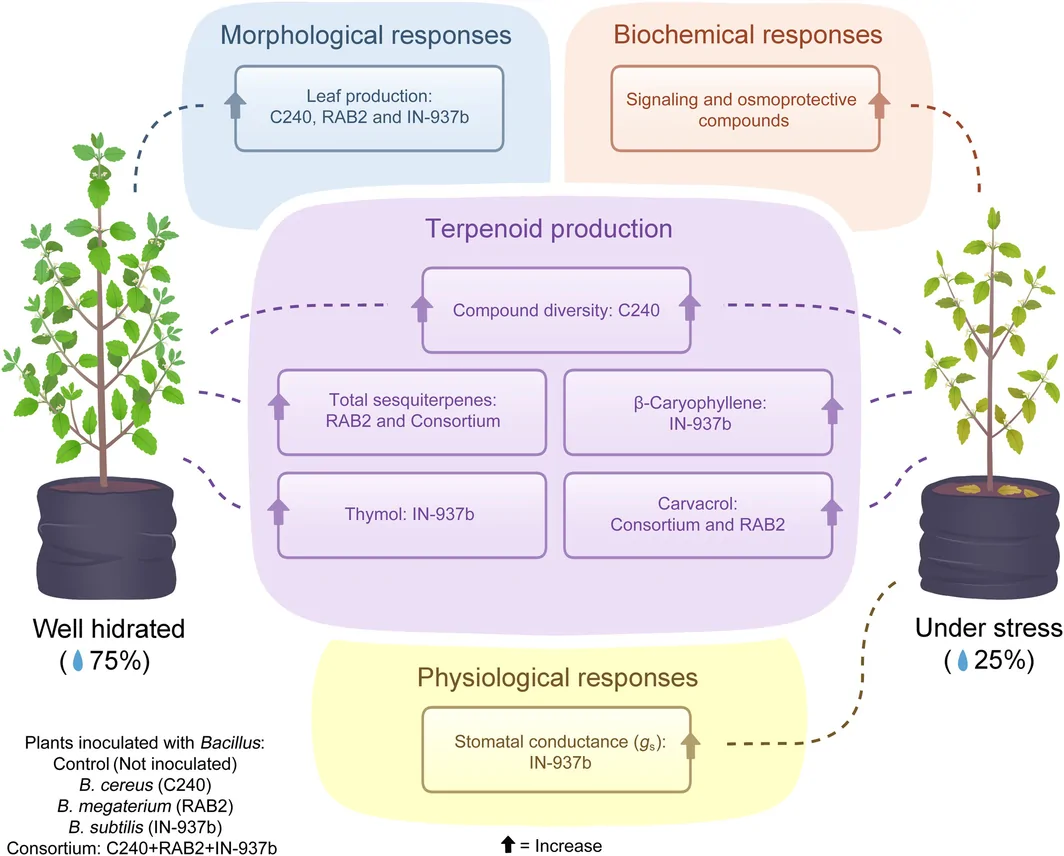
Main effects of different *Bacillus* inoculations: Control (non‐inoculated), *B. cereus* (C240), *B. megaterium* (RAB2), *B. subtilis* (IN‐937b), and a consortium of these strains under two water conditions, hydrated (75%) and water stress (25%), on morphological, physiological, biochemical parameters, and essential oil content of *Lippia origanoides* Kunth.

The experiment was conducted in a greenhouse, and biochemical analyses were carried out at the Plant Physiology Laboratory, both of which belong to the Department of Biology at the Federal Rural University of Pernambuco (UFRPE), in Recife, Pernambuco, Brazil (8°00′48.6″ S 34°56′59.9″ W). The plants used in this experiment were sourced from the Botanical Garden of Recife, Pernambuco, Brazil (8°04′35.9″ S 34°57′34.9″ W) (Figure 1).

### 2.1 Plant Material and Experimental Conditions

**FIGURE 2:**
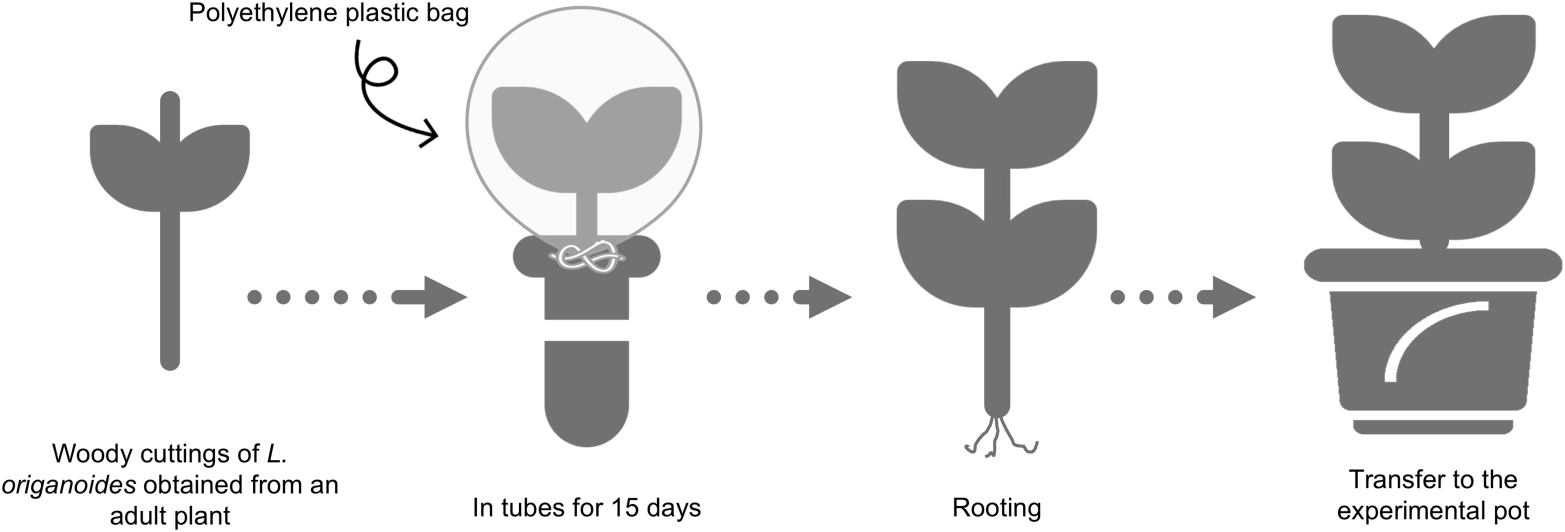
Production process of *Lippia origanoides* seedlings for the experiment.

Woody cuttings were collected from a single adult *L*. *origanoides* individual (approximately 20 cm in height and 0.5 cm in diameter), each containing two pairs of leaves, to ensure genetic uniformity. They were then planted in tubes filled with a 2:1 mixture of organic substrate and washed sand. After planting, the cuttings were covered with transparent plastic bags to maintain the humidity and promote rooting, facilitating their establishment and development. The bags were removed every 3 days to irrigate the cuttings via spraying. This procedure lasted approximately 15 days or until new leaf pairs emerged (Figure 2).

This mixture was previously sterilized in an autoclave at 120°C for 60 min, twice, on consecutive days. The substrate mixture exhibited the following chemical characteristics: pH 7.22, Ca, Mg, Al, Na, and K contents of 4.8, 0.0, 0.0, 0.26, and 0.15 cmolc dm^−3^, respectively; P content of 383.73 mg dm^−3^, and organic matter content of 35.93 g kg^−1^.

#### 2.1.1 Bacterial Strains, Culture Media and Conditions, and Experimental Treatments

Three bacterial strains used for inoculation were selected based on their potential to enhance plant growth: *Bacillus cereus* (strain C240), *Bacillus megaterium* (strain RAB2), and *Bacillus subtilis* (strain IN‐937b). These strains were provided by the Phytobacteriology Laboratory of UFRPE. The bacterial strains were cultured in nutrient broth for isolation and propagation (Luria‐Bertani medium supplemented with 10 g L^−1^ tryptone, 5 g L^−1^ yeast extract, and 10 g L^−1^ NaCl) and maintained in nutrient broth with 20% glycerol. Individual colonies were transferred to 100‐mL flasks and incubated aerobically in a rotary shaker at 200 rpm for 48 h at 28°C. The bacterial suspension was diluted in sterilized distilled water to a final concentration of 10^9^ colony‐forming units (CFUs) per milliliter for inoculation (Santoro et al. 2011).

After the plants were transferred to the experimental pots, a single application of the bacterial suspension (50 mL) was applied to the soil near the roots, and the non‐inoculated control plants received 50 mL of sterile distilled water. The experiment included five treatments involving bacterial inoculation with *Bacillus* species, all based on bacterial suspensions: (1) control: no inoculation; (2) inoculation with *B. cereus* (strain C240); (3) inoculation with *B. megaterium* (strain RAB2); (4) inoculation with *B. subtilis* (strain IN‐937b); and (5) a mixture containing equal concentrations of *B. cereus* , *B. megaterium* , and *B. subtilis* .

The experiment lasted 70 days. At the beginning, the plants received a single soil application of the bacterial suspension. At 33 days after inoculation, they were subjected to two water regimes based on vessel capacity: hydrated plants (75%) and water‐stressed plants (25%). The soil moisture was monitored and adjusted daily using a portable soil moisture sensor (HydroSense II, Campbell Scientific Inc., Logan, Utah, USA), which determines the volumetric water content (VWC). Based on prior calibration, the 75% and 25% irrigation levels corresponded to approximately 19.7% and 6.6% VWC, respectively. The plants were maintained under these irrigation regimes for 37 days.

**FIGURE 3:**
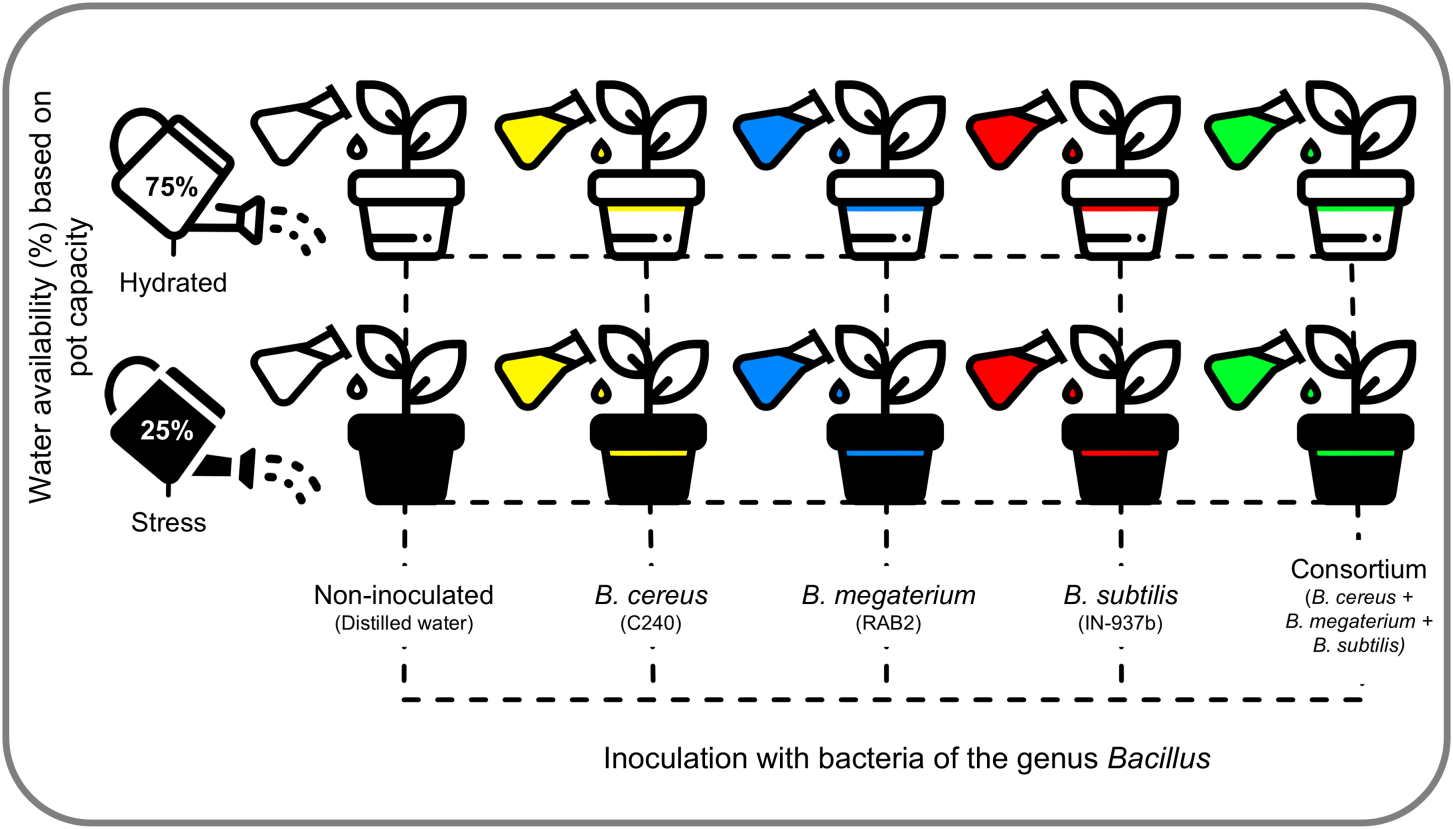
Experimental setup of co‐cultivation with plant growth‐promoting bacteria of the genus *Bacillus* ( *B. cereus* C240, *B. megaterium* RAB2 and *B. subtilis* IN‐937b) *in Lippia origanoides* plants subjected to treatments with different water availability levels: 75% (Hydrated) and 25% (Stress).

The experiment was conducted using a completely randomized design in a 5 × 2 factorial arrangement (5 bacterial inoculation treatments × 2 water conditions), resulting in 10 experimental combinations, with 7 replicates per treatment, for a total of 70 experimental plants (Figure 3).

### 2.2 Analysis and Measurements

#### 2.2.1 Growth Analyses

The stem growth rate (measured as the distance between the base of the plant and the most recently fully developed node) and leaf production were assessed using 7 replicates per treatment (*n* = 7). Measurements were taken at 16, 40, 54, and 68 days after bacterial inoculation. Growth analysis was conducted by calculating the difference (Δ) between the values at the different time intervals (from the first to the second (*time interval 1*), from the second to the third (*time interval 2*), and from the third to the fourth (*time interval 3*)) using the following formula:

∆=Pf−Pi

where ‘Pi’ represents the initial value and ‘Pf’ represents the final value. The formula calculates the variation between the final and initial values over a given time interval, reflecting the change or growth that occurred during this period.

The ratio between leaves and flowers (inflorescences) was assessed using 3 biological replicates per treatment (*n* = 3) at 68 days, corresponding to the minimum number of flowering plants with inflorescences in all treatments. In treatments with more than three individuals with inflorescences, three replicates were selected to standardize the sample size for comparison. As flowering did not occur in all treatments, the 75% water treatment without bacterial inoculation was excluded due to the absence of flowering individuals. The ratio was calculated using the following formula:

$$\text{Leaf}-\text{to}-\text{Flower}-\text{Stalk Ratio}=\frac{\text{Total number of leaves}}{\text{Total number of flower stalks}}$$

#### 2.2.2 Physiological Analyses

Stomatal conductance (*g*_s_) was measured using a leaf porometer (SC‐1 Leaf Porometer, METER Group Inc.), and the total chlorophyll content was measured using a chlorophyll meter (SPAD‐502Plus, Konica Minolta Sensing Inc.), taking an average of 5 readings per leaf. Measurements were taken in the early morning (6–9 AM) on the youngest fully expanded healthy leaf of four individuals per treatment. Measurements were performed on 4 individuals per treatment (*n* = 4), selected to enable simultaneous evaluation under consistent environmental conditions. The relative humidity and average temperature during the experimental period (June–September 2023) are shown in Table 1.

**TABLE 1:** Relative humidity (%) and mean temperature (°C) during the experimental period (June–September 2023).

Evaluation times	Days after bacterial inoculation	Relative humidity (%)	Temperature (°C)
*Time* 1	19	77	26.5
*Time* 2	41	78	28.5
*Time* 3	55	80	28
*Time* 4	69	70	29.5

*Note:* Relative humidity and temperature correspond to the average values recorded at the beginning and end of the physiological measurements (stomatal conductance and SPAD index) for each evaluation period.

For the biochemical and EO analyses described below, 4 biological replicates per treatment (*n* = 4) were used, corresponding to the subset of plants allocated to these analyses within the experimental design.

#### 2.2.3 Biochemical Analyses

Leaves from the middle region of each plant, which were healthy, fully expanded, and without signs of senescence, and young, fine roots were collected. Immediately after harvest, the material was frozen in liquid nitrogen and stored at −20°C until biochemical analysis. Leaf and root tissues were homogenised in liquid nitrogen, and absorbance measurements were performed using a UV–Vis single‐beam spectrophotometer (M‐51, Bel Photonics).

A total of 100 mg of fresh leaf weight and 100 mg of fresh root weight were used. The samples were homogenised in liquid nitrogen and prepared with 12,000 μL of 80% ethanol. The extract was centrifuged at 5500 *g* for 10 min. Aliquots of this extract were collected to determine the photosynthetic pigment, soluble carbohydrate, and total free amino acid contents.

The crude ethanolic extract was used to determine the chlorophyll *a* and *b* and carotenoid contents. Absorbance was measured at 663, 645, and 470 nm for chlorophyll *a* and *b* and carotenoid contents. The pigment contents were calculated according to Lichtenthaler and Buschmann (2001), and the results were expressed in g kg^−1^ dry weight and for carotenoids in μg mL.

The soluble carbohydrate content was measured using 100 μL of ethanolic extract (leaf or root), to which 2000 μL of the anthrone reagent (0.2%) was added. The reaction was incubated in a water bath at 100°C for 10 min and then immediately cooled in an ice bath for 5 min. Absorbance was measured at 620 nm, and the results were expressed in mg g^−1^ dry weight (Yemm and Willis 1956).

The total free amino acid content was measured using a reaction consisting of 250 μL of 200 mM citrate buffer (pH 5), 600 μL of the reagent (potassium cyanide at 0.2 mM in methylcellosolve and 5% ninhydrin), and 250 μL leaf extract or 500 μL root extract. The reaction was subjected to a hot bath at 100°C for 15 min and rapidly cooled in an ice bath for 5 min, and then 1500 μL of 60% ethanol was added. The absorbance was read at 620 nm. The results were expressed as mg g^−1^ dry weight (Yemm et al. 1955).

For proline extraction, 20 mg of fresh leaf mass and 40 mg of fresh root weight were used. The extract was prepared in 3% sulfosalicylic acid and centrifuged at 5500 g for 10 min. For the reaction, 1000 μL of the extract, 1000 μL of acid ninhydrin (prepared with glacial acetic acid and 6 M phosphoric acid), and 1000 μL of glacial acetic acid were used. The reaction was placed in a hot water bath at 100°C for 1 h and rapidly cooled in an ice bath for 5 min. Subsequently, 2000 μL of toluene was added, followed by 20 s of shaking and a waiting period of 5–10 min for phase separation. Absorbance was measured at 520 nm, and the results were expressed in μmol g^−1^ dry weight (Bates et al. 1973).

To extract the total soluble protein content, 200 mg of fresh leaf tissue was used, and 2000 μL of 100 mM sodium phosphate buffer (pH 7.5) supplemented with EDTA, dithiothreitol, and 40 mg of polyvinylpyrrolidone (PVP), an antioxidant agent, were added. The material was centrifuged at 5500 g for 30 min at −4°C, and the supernatant was used to determine the total soluble protein content according to the Bradford (1976) method and the activity of the antioxidant system enzymes. The results were expressed as mg g^−1^ dry weight.

The plant extract used for the enzymatic activity analyses was the same as that used in the extraction of the total soluble protein content, as described earlier.

The SOD activity (E.C. 1.15.1.1) was determined in a reaction consisting of 1765 μL of 85 mM sodium phosphate buffer (pH 7.8), 780 μL of 13 mM methionine, 225 μL of 75 mM nitro blue tetrazolium, 30 μL of 0.1 mM EDTA, 150 μL of 5 μM riboflavin, and 50 μL of plant extract. The reaction was placed in glass tubes and exposed to fluorescent light (15 W) for 5 min. Absorbance was measured at 560 nm (Giannopolitis and Ries 1977), and the activity was expressed as SOD units per mg of protein.

The CAT activity (E.C. 1.11.1.6) was analyzed with 3 replicates per sample using a reaction composed of 1390 μL of 100 mM potassium phosphate buffer (pH 7.5), 50 μL of plant extract, and 60 μL of 100 mM H_2_O_2_. H_2_O_2_ decomposition was monitored by the decrease in absorbance, which was measured at 240 nm for 1 min (Azevedo et al. 1998; Havir and McHale 1987). The activity was expressed as mmol H_2_O_2_ mg^−1^ protein min^−1^.

The APX activity (E.C: 1.11.1.11) was measured in 3 replicates per sample, using a reaction consisting of 650 μL of 80 mM potassium phosphate buffer (pH 7), 100 μL of EDTA, 50 μL of the plant extract, 100 μL of 5 mM ascorbate, and 100 μL of 1 mM H_2_O_2_. The APX activity was monitored by the rate of ascorbate oxidation for 1 min in a spectrophotometer at 290 nm (Nakano and Asada 1981). The activity was expressed in mmol ASA mg^−1^ protein min^−1^.

To extract MDA and H_2_O_2_, 200 mg of fresh leaf and root tissue were used, to which 2000 μL of 0.1% trichloroacetic acid supplemented with polyvinylpyrrolidone was added. The material was centrifuged at 5500 g for 5 min at −4°C, and the supernatant was used to determine the MDA and H_2_O_2_ contents.

The MDA content was determined by the reaction of 250 μL of plant material (leaf or roots) with 1000 μL of 20% trichloroacetic acid and 0.5% thiobarbituric acid. The reaction was heated in a water bath at 100°C for 30 min, cooled in an ice bath for 10 min, and then centrifuged at 5500 g for 10 min. The absorbance was measured at 535 and 600 nm (Heath and Packer 1968), and peroxidation was expressed in μmol g^−1^ of dry weight.

For the H_2_O_2_ reaction, 200 μL of plant material (leaf or roots), 800 μL of 1000 mM potassium iodide, and 200 μL of 100 mM potassium phosphate buffer (pH 7.5) were used and incubated for 1 h in the dark. The absorbance was measured at 390 nm (Loreto and Velikova 2001). The content was expressed in μmol g^−1^ of dry weight.

#### 2.2.4 Quantitative and Qualitative Analysis of Essential Oil

EO extraction was performed via steam distillation using a Clevenger‐type apparatus, employing 3–30 g of fresh leaf biomass per sample. The leaves were weighed, ground, and placed in a round‐bottom flask containing water. The distillation process was carried out for 2 h at 100°C. The essential oil yield (EOY) was calculated using the following formula:

$$\mathrm{EOY}\;\left(\%\right)=\frac{W\;\left(\text{oil}\right)}{W\left(\text{leaves}\right)}\times 100$$

where “EOY (%)” indicates the essential oil yield in percentage; “W (oil)” represents the oil weight in grams; and “W (leaves)” refers to the fresh leaf weight in grams.

Gas chromatography–mass spectrometry (GC–MS) analysis was carried out using a GC‐EM QP2010 SE Plus system (Shimadzu Corporation) with a mass selective detector, a mass spectrometer in EI 70 eV with a scan‐interval of 0.5 s, and fragments from 40 to 550 Da. Non‐polar fused silica capillary column DB‐5 (30 × 0.25 × 0.25 mm) was used. The oven temperature was programmed to increase from 60°C to 240°C at a rate of 3°C min^−1^. The injector and detector temperatures were set at 260°C. Helium was used as the carrier gas at a flow rate of 1 mL min^−1^ in split mode (1:30). The volume injected in auto‐injector AOC6000 was 1 mL of a diluted solution (1:100) of oil in n‐hexane.

Components were identified based on GC–MS retention indices with reference to a homologous series of C8–C40 n‐alkanes calculated using the Van den Dool and Kratz equation (Van Den Dool and Kratz 1963) by comparison against the mass spectral library of the GC–MS data system (NIST 14 and WILEY 14th) and co‐injection of authentic standards as well as other published mass spectra (Adams 2017). Area percentages were obtained from the GC–MS response using an internal standard for correction factors. All standards were purchased from Sigma‐Aldrich.

#### 2.2.5 Statistical Analysis

To determine whether bacterial inoculation (predictor variable) affected the physiological parameters of stomatal conductance (*g*_s_) and the SPAD index (total chlorophyll) at *time* 1, the assumptions of normality and homoscedasticity were checked, and one‐way analysis of variance (ANOVA) with Tukey's HSD test (*p* ≤ 0.05) was performed for mean comparisons.

To determine whether bacterial inoculation and water treatment influenced the growth parameters, physiological parameters (at *times* 2, 3, and 4), biochemical parameters (photosynthetic pigment, total soluble carbohydrate, total free amino acid, total soluble protein, proline, antioxidant enzyme, and stress marker contents), and EOY at the end of the experimental period, the assumptions of normality and homoscedasticity were tested, and a two‐way ANOVA was performed. When the residuals did not follow a normal distribution (leaf/flower ratio, chlorophyll *a*, chlorophyll *b*, total chlorophyll, proline content in leaves, total soluble protein content, amino acid content in roots, MDA content in roots, H_2_O_2_ content in leaves, and CAT and APX activities), cubic root transformations were applied. Post hoc comparisons were performed using Tukey's test (*p* ≤ 0.05) with the *ExpDes*.*pt* package (Ferreira et al. 2014).

For variables that did not follow a normal distribution even after transformation (SPAD index and SOD), a non‐parametric two‐way ANOVA was conducted using the aligned rank transformation method in the *ARTool* package (Wobbrock et al. 2011). For post hoc comparisons of these variables, the Bonferroni adjustment (*p* ≤ 0.05) was applied using the *contrast* and *cld* functions from the *emmeans* and *multcomp* packages. All analyses were conducted using R software (version 4.4.1).

## 3 Results

### 3.1 Growth Parameters, Stomatal Conductance, and SPAD Index

A significant interaction was observed between water availability and bacterial inoculation in leaf production during the first time interval (Table S1). Compared to stressed plants under the same treatments, well‐irrigated plants showed increases of 88% with *B. cereus* (C240), 60% with *B. megaterium* (RAB2), and 40% with *B. subtilis* (IN‐937b) (Table 2). Considering only the well‐irrigated treatments, *B. cereus* (C240) resulted in 77% higher leaf production compared to plants that did not receive bacterial inoculation.

**TABLE 2:** Leaf production, relative growth, and leaf‐to‐flower ratio in *Lippia origanoides* Kunth subjected to bacterial inoculation with *Bacillus* species: Control (non‐inoculated), *B. cereus* (C240), *B. megaterium* (RAB2), *B. subtilis* (IN‐937b), and a consortium composed of these strains, under different water availability conditions: 75% (Hydrated) and 25% (Stress), evaluated across four experimental periods.

Inoculation with bacteria of the genus *Bacillus*	*Time interval* 1		*Time interval* 2		*Time interval* 3	
	Leaf production					
	Hydrated	Stress	Hydrated	Stress	Hydrated	Stress
Non‐inoculated	84.14 ± 7.87 aB	79.00 ± 7.08 aA	117.71 ± 10.60 aA	33.00 ± 13.99 aB	132.86 ± 40.70 aA	95.71 ± 16.90 aB
*B. cereus* (C240)	149.29 ± 12.99 aA	79.29 ± 9.61 bA	121.29 ± 16.00 aA	33.71 ± 10.61 aB	164.00 ± 23.77 aA	40.00 ± 10.53 aB
*B. megaterium* (RAB2)	110.57 ± 7.15 aB	68.71 ± 7.49 bA	138.29 ± 14.56 aA	35.14 ± 6.82 aB	178.43 ± 37.78 aA	67.86 ± 18.29 aB
*B. subtilis* (IN‐937b)	94.71 ± 8.65 aB	67.29 ± 8.31 bA	121.43 ± 7.24 aA	40.86 ± 16.23 aB	167.57 ± 46.97 aA	33.57 ± 9.21 aB
Consortium	84.57 ± 11.55 aB	91.43 ± 7.35 aA	174.43 ± 23.16 aA	37.57 ± 10.03 aB	189.86 ± 48.68 aA	25.14 ± 6.40 aB

Inoculation with bacteria of the genus *Bacillus*	Stem growth					
	Hydrated	Stress	Hydrated	Stress	Hydrated	Stress
Non‐inoculated	39.09 ± 1.70 aA	36.84 ± 2.07 aB	24.94 ± 0.81 aA	17.67 ± 2.15 aB	14.36 ± 0.74 aA	5.29 ± 0.87 bB
*B. cereus* (C240)	33.55 ± 2.80 bA	27.60 ± 2.50 bB	25.77 ± 0.69 aA	9.30 ± 1.76 aB	15.99 ± 1.30 aA	5.14 ± 0.55 bB
*B. megaterium* (RAB2)	36.69 ± 1.78 abA	30.67 ± 2.89 abB	23.39 ± 2.98 aA	10.09 ± 2.18 aB	18.50 ± 2.06 aA	5.21 ± 1.27 bB
*B. subtilis* (IN‐937b)	32.81 ± 2.05 bA	25.99 ± 2.46 bB	24.19 ± 0.99 aA	13.51 ± 2.21 aB	19.36 ± 1.36 aA	4.14 ± 0.90 bB
Consortium	34.67 ± 2.84 abA	33.87 ± 3.08 abB	24.83 ± 3.14 aA	12.06 ± 2.21 aB	14.00 ± 1.70 aA	5.43 ± 0.90 bB

Inoculation with bacteria of the genus *Bacillus*	Leaves/flowers			
		Hydrated	Stress	
Non‐inoculated		There was no flowering	1.68 ± 0.07 aB	
*B. cereus* (C240)		1.96 ± 0.048 aA	1.33 ± 0.16 aB	
*B. megaterium* (RAB2)		2.19 ± 0.09 aA	1.40 ± 0.27 aB	
*B. subtilis* (IN‐937b)		3.06 ± 0.6 aA	1.34 ± 0.19 aB	
Consortium		3.06 ± 0.45 aA	1.17 ± 0.01 aB	

*Note:* Statistical significance was determined using Tukey's test at a significance level of *p* ≤ 0.05. The table presents the means ± standard error. Leaf production (*n* = 7), relative growth (*n* = 7), and the leaf‐to‐flower ratio (*n* = 3) were evaluated. Lowercase letters indicate differences between bacterial inoculation treatments (single factor) or under different water conditions (interaction between bacterial inoculation and water condition), whereas uppercase letters indicate differences within water treatment conditions (single factor). See Supporting Information.

During the first time interval, plant growth was influenced by bacterial inoculation, regardless of the water availability. Plants treated with *B. cereus* (C240) and *B. subtilis* (IN‐937b) exhibited reduced growth compared to non‐inoculated plants. However, plants inoculated with *B. megaterium* (RAB2) and the bacterial consortium did not statistically differ from the non‐inoculated plants (Table 2).

In subsequent periods, leaf production, relative growth, and the ratio between leaves and flowers (assessed only at the end of the experiment) only showed statistical differences based on water availability. These parameters were higher under increased irrigation compared to the lower irrigation treatments (Table 2).

**FIGURE 4:**
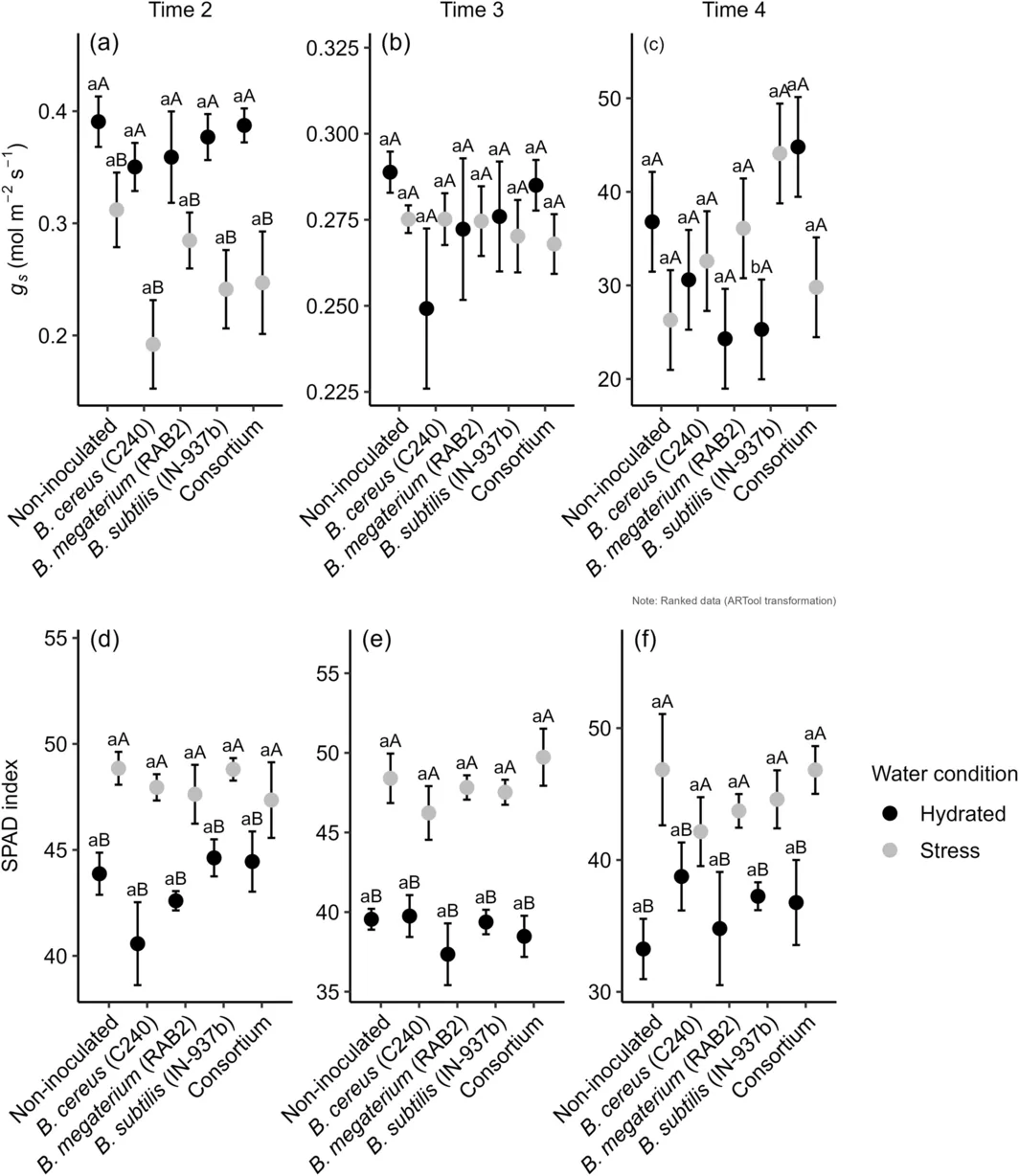
Stomatal conductance (*g*_s_; a–c) and total chlorophyll content (SPAD index; d–f) of *Lippia origanoides* Kunth subjected to bacterial inoculation with strains of the genus *Bacillus*: Control (non‐inoculated), *B. cereus* (C240), *B. megaterium* (RAB2), *B. subtilis* (IN‐937b), and a consortium composed of these bacterial strains. Evaluations at times 2, 3, and 4 represent the effects of bacterial inoculation combined with different water availability treatments: 75% (Hydrated) and 25% (Stress). Statistical significance was determined using Tukey's test or Bonferroni correction at *p* ≤ 0.05. Data are presented as means ± standard error (*n* = 4). For *g*_s_ at time 4, ranked values obtained by ARTool transformation were used for statistical analysis, whereas the original untransformed biological values are provided in Table S9. Lowercase letters indicate differences among bacterial inoculation treatments under different water conditions (interaction between inoculation and water availability), whereas uppercase letters indicate differences within water treatments (single factor). See Supporting Information.

Stomatal conductance (*g*_s_) at *time* 1 did not show significant differences (Table S2). At *time* 2, only the water factor resulted in significant differences (Figure 4a). Plants that received a higher amount of water exhibited a significantly higher *g*_s_ compared to those subjected to lower water availability, regardless of bacterial inoculation. However, at *time* 3, no significant differences were observed (Figure 4b). At *time* 4, a significant interaction between the factors was observed but only in the treatment with *B. subtilis* (IN‐937b), which showed a higher *g*_s_ under lower water availability (Figure 4c).

The total chlorophyll levels (SPAD) measured over time showed no significant differences at *time* 1 (Table S2). However, at *times* 2 (Figure 4d), 3 (Figure 4e), and 4 (Figure 4f), plants subjected to lower water availability had a significantly higher total chlorophyll index compared to well‐hydrated plants.

### 3.2 Components of Primary Metabolism

**FIGURE 5:**
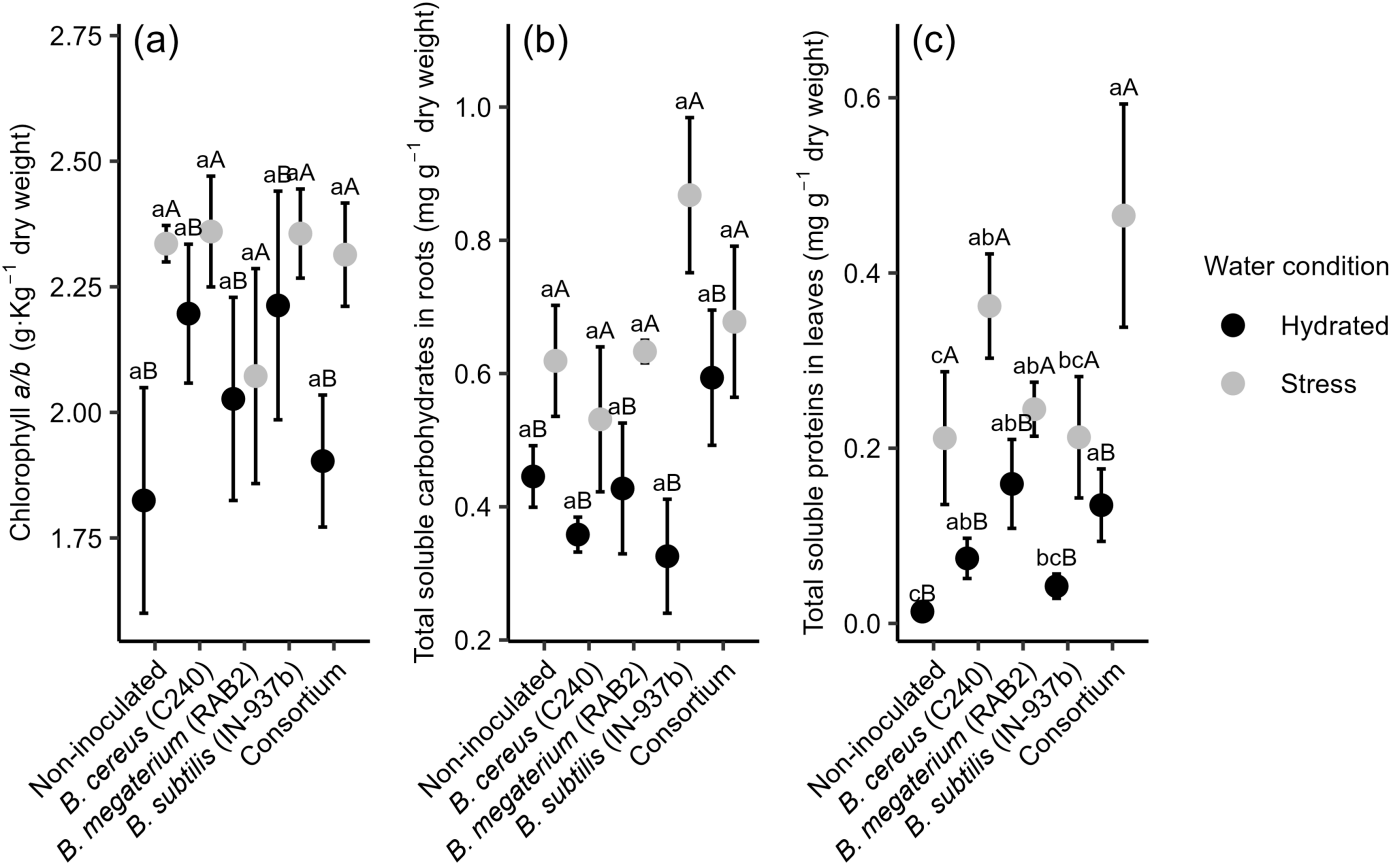
Chlorophyll *a/b* content (a), total soluble carbohydrates in roots (b), and total soluble proteins in leaves (c) of *Lippia origanoides* Kunth subjected to co‐cultivation with bacterial inoculants of the *Bacillus* genus: Control (non‐inoculated), *B. cereus* (C240), *B. megaterium* (RAB2), *B. subtilis* (IN‐937b), and a consortium composed of equal proportions of the three bacterial inoculants under different water availability treatments: 75% (Hydrated) and 25% (Stress), after 37 days of co‐cultivation. Statistical significance was determined using Tukey's test at *p* ≤ 0.05. Graphs represent mean values ± standard error (*n* = 4). Lowercase letters indicate differences among bacterial inoculation treatments under different water availability conditions (interaction between inoculation and water availability), whereas uppercase letters indicate differences within water treatments (single factor). See Supporting Information.

The chlorophyll *a*, chlorophyll *b*, total chlorophyll, carotenoid, and soluble carbohydrate contents in the leaves did not differ significantly between the water availability and bacterial treatments, either individually or interactively (Tables S3 and S4). However, plants subjected to lower water availability exhibited a higher chlorophyll *a*/*b* ratio (Figure 5a), increased soluble carbohydrate content in the roots (Figure 5b), and greater total soluble protein accumulation in the leaves (Figure 5c). Moreover, inoculated plants, except those treated with *B. subtilis* , showed a higher leaf protein content compared to non‐inoculated plants, regardless of the hydration level.

**FIGURE 6:**
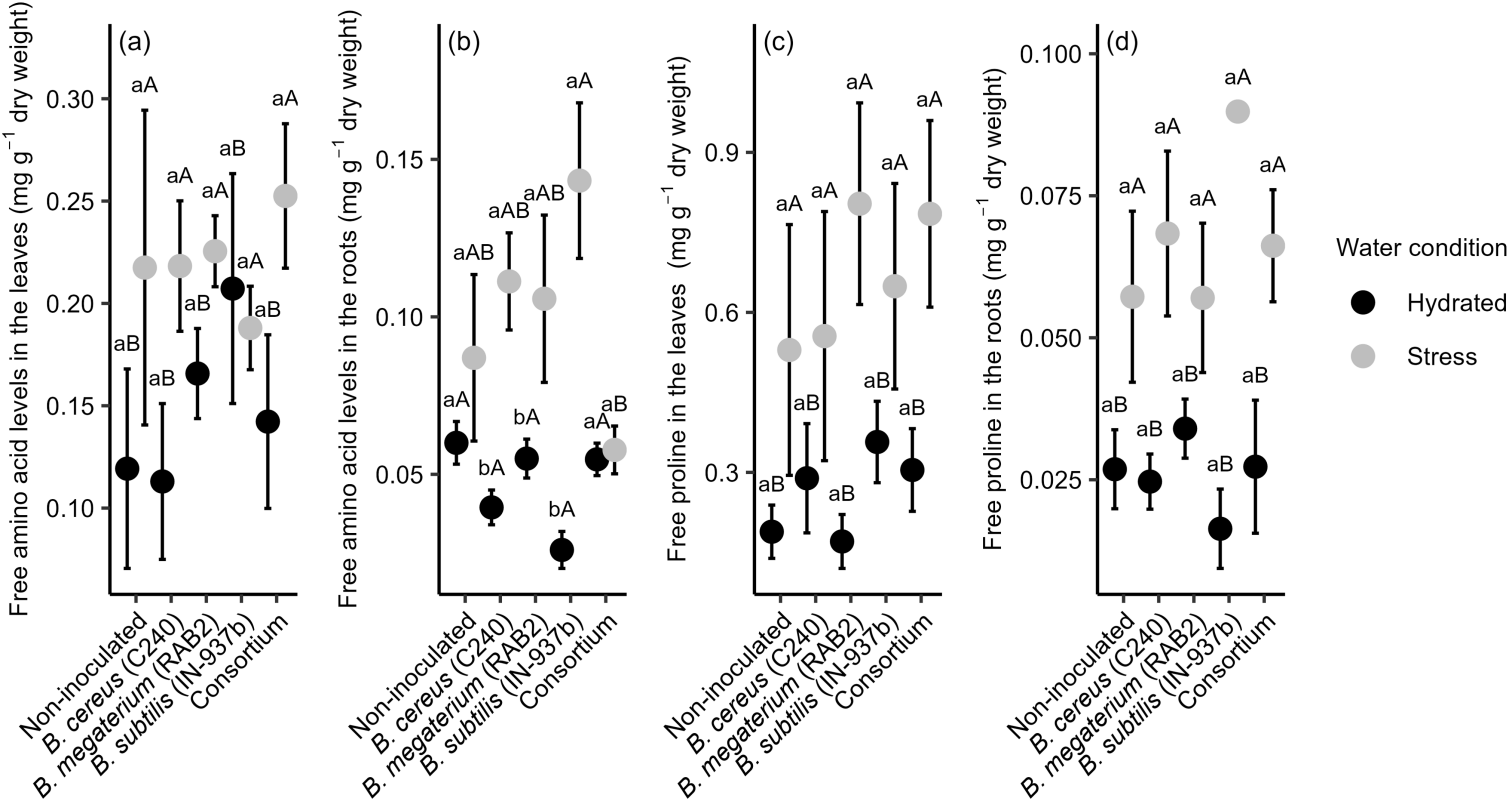
Free amino acid levels in the leaves (a), amino acid levels in the roots (b), free proline content in the leaves (c), and free proline content in the roots (d) of *Lippia origanoides* Kunth subjected to co‐cultivation with bacterial inoculants of the *Bacillus* genus: Control (non‐inoculated), *B. cereus* (C240), *B. megaterium* (RAB2), *B. subtilis* (IN‐937b), and a consortium composed of equal proportions of the three bacterial inoculants under different water availability treatments: 75% (Hydrated) and 25% (Stress), after 37 days of co‐cultivation. Statistical significance was determined using Tukey's test at *p* ≤ 0.05. Data are presented as means ± standard error (*n* = 4). Lowercase letters indicate differences among bacterial inoculant treatments under different water conditions (interaction between inoculant and water condition), whereas uppercase letters indicate differences within water availability conditions (single factor). See Supporting Information.

The total free amino acid (Figure 6a) and free proline contents in the leaves (Figure 6c) and roots (Figure 6d) varied significantly as a function of water availability with the highest values observed in the plants subjected to water stress. Water‐stressed plants inoculated with individual bacterial strains showed a higher free amino acid content in the roots compared to well‐hydrated plants (Figure 6b).

### 3.3 Markers of Stress and Enzymes of the Antioxidant System

**FIGURE 7:**
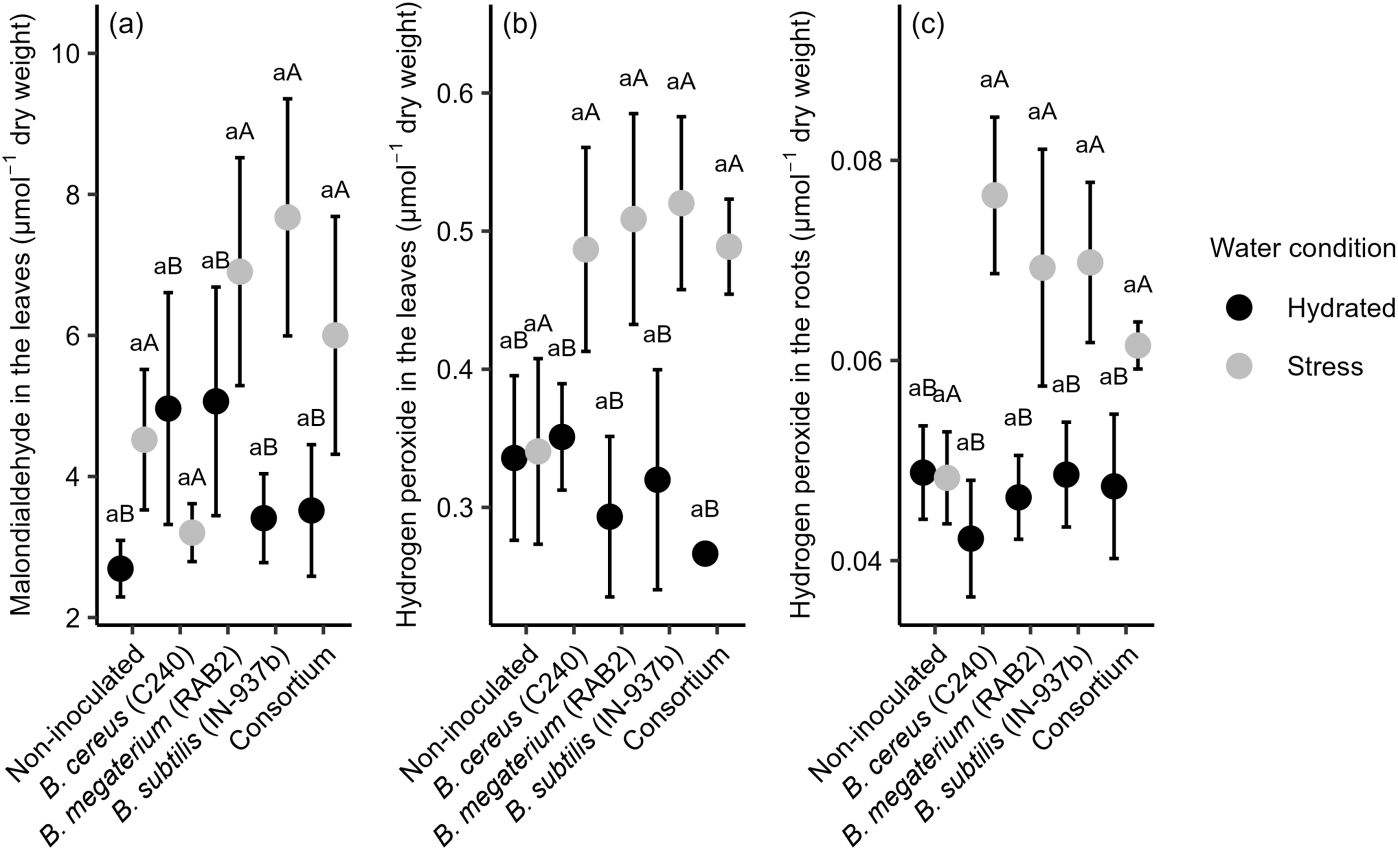
Malondialdehyde (MDA) levels in the leaves (a), hydrogen peroxide (H_2_O_2_) levels in the leaves (b), and hydrogen peroxide (H_2_O_2_) levels in the roots (c) of *Lippia origanoides* Kunth subjected to co‐cultivation with bacterial inoculants of the *Bacillus* genus: Control (non‐inoculated), *B. cereus* (C240), *B. megaterium* (RAB2), *B. subtilis* (IN‐937b), and a consortium composed of equal proportions of the three bacterial inoculants under different water availability treatments: 75% (Hydrated) and 25% (Stress), after 37 days of co‐cultivation. Statistical significance was determined using Tukey's test at *p* ≤ 0.05. Data are presented as means ± standard error (*n* = 4). Lowercase letters indicate differences among bacterial inoculant treatments under different water conditions (interaction between inoculant and water condition), whereas uppercase letters indicate differences within water availability treatments (single factor). See Supporting Information.

The MDA content in the roots did not show a significant difference, either in isolation or in the interaction between factors (Table S5). However, the MDA content in the leaves (Figure 7a) and H_2_O_2_ content in the leaves (Figure 7b) and roots (Figure 7c) exhibited significant differences considering water availability. Under a lower water availability, the plants had higher MDA and H_2_O_2_ contents.

**FIGURE 8:**
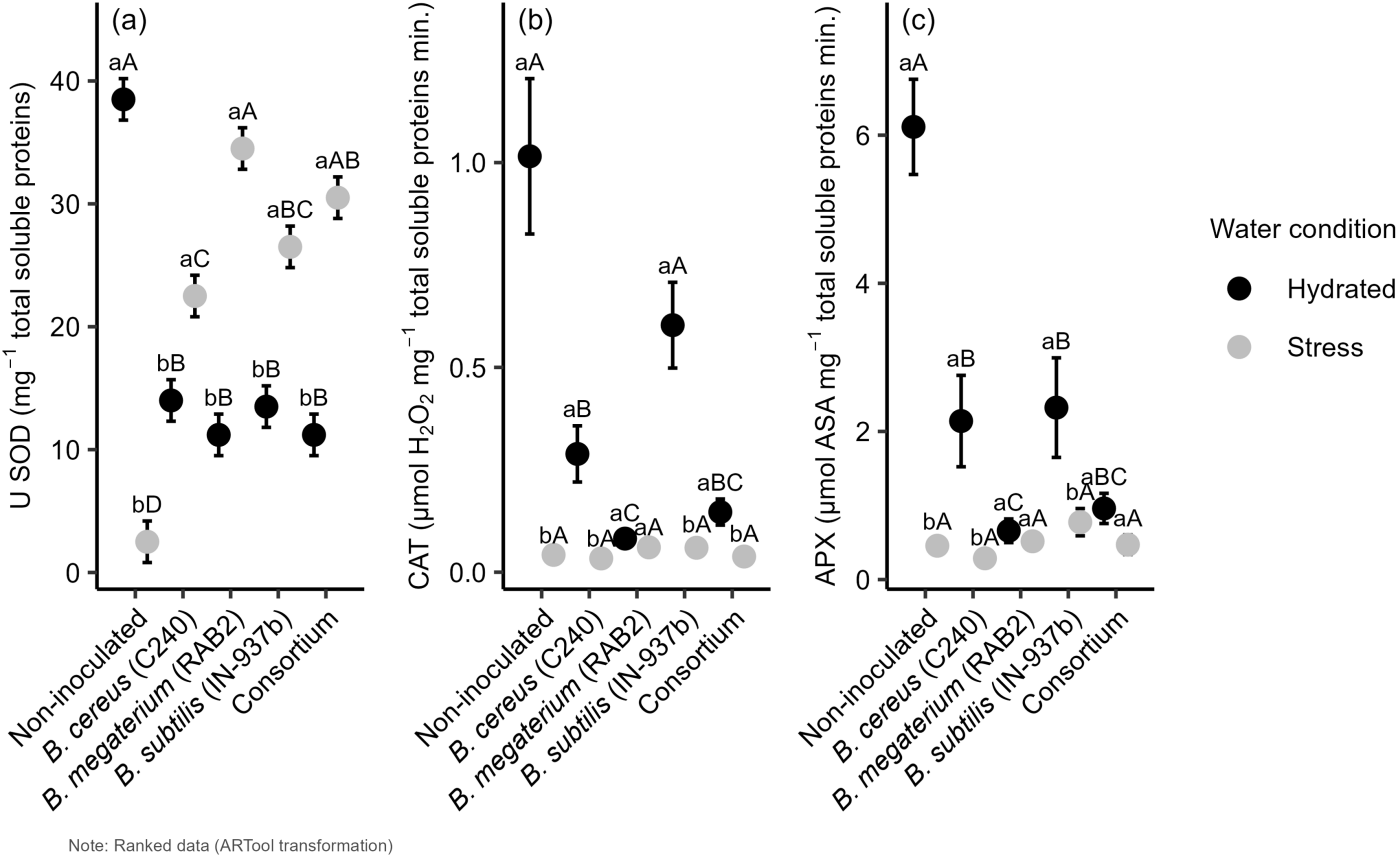
Activity of antioxidant enzymes: Superoxide dismutase (SOD) (a), catalase (CAT) (b), and ascorbate peroxidase (APX) (c) in *Lippia origanoides* Kunth subjected to co‐cultivation with bacterial inoculants of the *Bacillus* genus: Control (non‐inoculated), *B. cereus* (C240), *B. megaterium* (RAB2), *B. subtilis* (IN‐937b), and a consortium composed of equal proportions of the three bacterial inoculants under different water availability treatments: 75% (Hydrated) and 25% (Stress), after 37 days of co‐cultivation. Statistical significance was determined using Tukey's test at *p* ≤ 0.05. Data are presented as means ± standard error (*n* = 4). For SOD, the figure presents ranked values obtained by ARTool transformation and used for statistical analysis, whereas the original untransformed biological values are provided in Table S10. Lowercase letters indicate differences among bacterial inoculant treatments under different water availability conditions (interaction between inoculant and water condition), whereas uppercase letters indicate differences within water availability conditions (single factor). See Supporting Information.

The enzyme activities of SOD (Figure 8a), CAT (Figure 8b), and APX (Figure 8c) exhibited a significant interaction between water availability and bacterial inoculation (Table S6). Plants subjected to a lower water availability and inoculated with bacteria displayed a higher SOD activity. The CAT and APX activities were generally higher under well‐hydrated conditions than under water‐stressed conditions. However, the CAT activity in plants inoculated with *B. megaterium* (RAB2) and the APX activity in plants inoculated with *B. megaterium* (RAB2) and the bacterial consortium showed no significant differences among inoculation treatments (Figure 8).

### 3.4 Quantitative and Qualitative Analysis of Essential Oil

EOY did not differ significantly among treatments (Table S7). A total of 32 compounds were identified, 11 of which were exclusive to bacterial inoculation treatments (Table S8). The EO profile was dominated by terpenes, particularly monoterpenes (carvacrol and thymol) and sesquiterpenes (β‐caryophyllene).

Water availability strongly influenced the terpenoid composition. Under water stress, inoculated plants, especially those treated with the bacterial consortium and *B. megaterium* (RAB2), showed a higher carvacrol content compared to those under well‐watered conditions. In contrast, thymol was more abundant in well‐hydrated plants inoculated with *B. subtilis* (IN‐937b), reaching values up to 114% higher than those under stress.

A similar pattern was observed for sesquiterpenes, which were generally more abundant under well‐watered conditions, particularly in treatments inoculated with *B. megaterium* and the consortium. Conversely, the β‐caryophyllene content was higher under water stress in both control plants and those inoculated with *B. subtilis* , with increases of up to tenfold compared to well‐watered conditions.

Overall, well‐watered plants exhibited a greater chemical diversity, whereas stress conditions were associated with a more restricted profile. Among the treatments, *B. cereus* (C240) was associated with the highest metabolite diversity, regardless of water availability.

## 4 Discussion

This study on the influence of bacterial inoculation on *L*. *origanoides* under water‐limited conditions initially demonstrated that inoculation with three of the five bacterial strains significantly enhanced leaf production. Water availability remained the primary factor affecting the evaluated parameters. Although inoculation with *B. subtilis* (IN‐937b) was associated with higher stomatal conductance (*g*_s_) under water stress at the end of the experimental period, its effect was secondary compared to water availability, suggesting a complementary role in physiological adjustment. Water availability shaped resource allocation, as well‐hydrated plants favored growth and reproduction and water‐limited plants shifted metabolism toward defense and stress‐related compounds, a pattern reflected in the leaf‐to‐flower ratio and potentially influenced by bacterial inoculation. Supporting these responses, inoculated plants exhibited a higher leaf protein content and increased protective amino acids in the roots, which are important under water stress. Although EOY did not differ among treatments, *Bacillus* inoculation was associated with changes in the terpenoid profile and antioxidant and insecticidal properties, suggesting their potential relevance for biotechnological applications.

Our hypotheses are based on the premise that: (1) bacterial inoculation enhances the morphophysiological performance of *L*. *origanoides* under water stress by improving growth parameters, such as leaf production and plant size, and optimizing stomatal dynamics; and (2) the presence of bacterial inoculation influences the stress response of *L*. *origanoides* under water restriction, redirecting plant metabolism toward a more efficient defense mechanism that is less detrimental to growth. This influence is manifested by increased antioxidant enzyme activity and osmoregulator accumulation, which reduces stress‐induced damage; (3) the interaction between water stress and bacterial inoculation affects the production and chemical composition of *L*. *origanoides* EO, resulting in an increase in the yield and/or the concentration of key compounds linked to the plant's physiological response to stress.

Regarding our first hypothesis, the results indicate that the evaluated physiological parameters, *g*_s_ and the SPAD index, were more strongly influenced by water availability than bacterial inoculation. Nevertheless, inoculation with *B. subtilis* (IN‐937b) was associated with a higher *g*_s_ in plants under a lower water availability in the final period. The variation in the leaf‐to‐flower ratio suggests the reallocation of metabolic resources, observed both under water stress and in plants inoculated with *Bacillus*. Therefore, both factors may contribute to changes in flowering patterns, possibly through distinct mechanisms. Under water stress, the anticipation of flowering occurs at the expense of vegetative growth, representing an adaptive strategy that enables the plant to complete its reproductive cycle before water deficit compromises survival (Verslues et al. 2023). This response is regulated by endogenous hormonal adjustments and the activation of florigenic genes that redirect metabolism to reproductive prioritization (Chen et al. 2023). Conversely, *Bacillus* inoculation may have promoted flowering through specific hormonal pathways, as bacteria of this genus modulate the synthesis and balance of phytohormones associated with reproductive processes (Fahad et al. 2015), stimulating flower formation even under adequate water conditions.

Other studies have suggested that plants undergo physiological and biochemical adjustments and morphological adaptations in response to water stress to cope with, escape, or tolerate oxidative stress (Rehman et al. 2024; Sandeep and Godi 2023; Wang et al. 2024; Wu et al. 2022). Physiological adjustments occur through controlled water loss via transpiration, which is primarily achieved by stomatal pore closure. Reduced irrigation directly lowers the soil water potential, decreasing the turgor pressure in plant cells. This condition restricts water uptake by the roots and induces stomatal closure in the leaves (Hemati et al. 2022). A lower *g*_s_ reduces water influx into cells, impairing division, elongation, differentiation, and expansion and limiting plant growth over time (Sandeep and Godi 2023).

Bacterial inoculation can support vegetative growth and enhance plant adaptation and abiotic stress tolerance (Cao et al. 2023; Tiepo et al. 2024). This effect may be associated with biofilm formation in the rhizosphere through exopolysaccharides (carbohydrate polymers), which strengthen interactions between bacteria and plant roots (Ajijah et al. 2023). Exopolysaccharides also improve the soil structure and stability, promoting greater water retention and nutrient availability for the plants (Abdelaal et al. 2021). Bacteria of the genus *Bacillus*, for instance, have been shown to stimulate vegetative growth and enhance physiological responses (Dodd et al. 2010). A similar pattern was observed in plants inoculated with *B. subtilis* (strain IN‐937b) under reduced water availability at the end of the experimental period.

In the first evaluated interval, foliar production was higher in the more hydrated plants inoculated with *B. cereus* (C240) than in the other equally hydrated treatments. Bacterial inoculation proved beneficial in this initial assessment; however, its effects were more pronounced in plants with a higher water availability. Under adequate hydration conditions, inoculation with *B. cereus* (C240) may have acted as a biofertiliser, stimulating phytohormone production and enhancing foliar growth due to its beneficial properties (Ferrusquía‐Jiménez et al. 2022). Although colonization was not directly assessed in the present study, the observed physiological and biochemical responses are consistent with plant‐bacteria interactions reported in the literature. However, this interpretation is constrained by the lack of microbiological confirmation.

Although inoculation with *B. cereus* (C240) positively influenced leaf production in the first time interval, this effect did not translate into increased overall vegetative growth. During this period, non‐inoculated plants were similar to those inoculated with *B. megaterium* (RAB2) and the bacterial consortium. Genomic analysis of *B. megaterium* (strain STB1) revealed genes associated with the uptake and transport of nitrogen compounds, such as nitrate and nitrite (Nascimento et al. 2020), which may contribute to the observed increase in leaf production. However, in this study, plants inoculated with *B. megaterium* (RAB2) allocated this investment toward stem structure (green stem) elongation rather than leaf production.

The second hypothesis, stating that plants subjected to water deficit reduce their growth rates but enhance defense responses, was confirmed. Under low water availability, the osmotic potential increased due to soluble carbohydrate accumulation in the roots as well as free amino acid and proline accumulation in both leaves and roots. The accumulation of these low‐molecular‐weight compounds serves as a biochemical strategy to protect plants from oxidative damage induced by water stress (Huang et al. 2023). Osmotic regulator accumulation can be facilitated by bacterial inoculants of the genus *Bacillus* (Gagné‐Bourque et al. 2016; Tiepo et al. 2018). In this study, bacterial inoculation was associated with increased osmotic compound accumulation in both leaves and roots. Regardless of water availability, inoculated plants showed a higher protein content in the leaves than non‐inoculated ones. Under water stress, plants may reduce the synthesis of some proteins and increase the production of defense‐related proteins that mitigate stress effects, particularly under more adverse conditions (Ahmad et al. 2019). In the roots, *Bacillus* inoculation was associated with higher concentrations of amino acids with protective functions, contributing to osmotic adjustment and metabolic stability under stress. These bacteria can induce the expression of drought‐related genes (Lastochkina 2021), reinforcing plant resilience under limited water conditions. Such biochemical responses may enhance the water potential gradient, improve turgor, protect cell membranes, and increase water uptake during water stress (Zarbakhsh and Shahsavar 2023).

Moreover, the increase in the chlorophyll *a* to *b* ratio and the SPAD index in plants under water stress suggests an adaptive response to maximize light capture. Under restricted water conditions, by reducing leaf production, as observed in this study, plants concentrate the chlorophyll content and indices in the remaining leaves, enhancing photosynthetic efficiency by minimizing self‐shading (Genesio et al. 2021). It is likely that energy resource reallocation and increased photosynthetic efficiency, such as chlorophyll content, are associated with the evolutionary strategy of species from tropical regions (Tiepo et al. 2024). This mechanism appears to be intrinsic to *L*. *origanoides* when subjected to a low water availability, regardless of bacterial inoculation.

Under severe stress, the biochemical mechanism of osmotic protection, provided by compounds, such as soluble carbohydrates and amino acids, may not be sufficient to mitigate the toxic effects of reduced water availability in the soil. Consequently, oxidative imbalance occurs due to the accumulation of ROS, such as singlet oxygen (^1^O_2_), superoxide anion (O_2_˙^−^), hydroxyl radical (OH˙^−^), and H_2_O_2_, in the leaves and roots (Wang et al. 2024). This imbalance can lead to desiccation and lipid peroxidation in the membranes, which is commonly assessed by measuring MDA. MDA is widely used as a marker of oxidative damage to chloroplasts and mitochondria, particularly in studies of abiotic stress and redox signaling (Abdelaal et al. 2021; Heath and Packer 1968). In association with bacterial inoculation, ROS and MDA contents may be reduced, as observed in plants treated with *Bacillus* strains under abiotic stress (Abd‐Allah et al. 2018). However, in the present study, bacterial inoculation treatments did not significantly influence the mitigation of stress markers. Nevertheless, plants subjected to reduced water availability exhibited an elevated MDA content in the leaves and H_2_O_2_ content in both leaves and roots, regardless of inoculation. Under severe stress, the effect of bacterial inoculation may have been insufficient to prevent membrane damage caused by oxidative stress.

Our second hypothesis proposed that bacterial inoculation would positively affect the tolerance of plants subjected to reduced water availability. This effect is mediated by the activation of plant enzymatic defense responses that mitigate ROS accumulation, alleviating stress and enhancing tolerance (Dodd et al. 2010). Such responses can involve the upregulation of antioxidant enzymes, including SOD, CAT, and APX, which reduce ROS levels (Dodd et al. 2010; Rukh et al. 2025). SOD serves as the primary enzymatic defense against oxidative stress by catalyzing the dismutation of O_2_˙^−^ into H_2_O_2_, which is detoxified by CAT and APX in response to stress (Pandey et al. 2017).

In this study, the highest SOD activity was observed in plants subjected to reduced water conditions and inoculated with bacteria, indicating an adaptive response to neutralize the damage caused by excess ROS generated under stress. Bacterial inoculation may contribute to redox homeostasis in plants by stimulating enzymatic antioxidant activity (Asghari et al. 2020), including SOD, CAT, and APX. These enzymes act synergistically to reduce ROS accumulation, protecting plant cells from oxidative damage and enhancing resilience under abiotic stress (Vázquez‐Hernández et al. 2019). However, an imbalance was observed, as CAT and APX did not follow the same pattern as SOD in stressed plants, except for those inoculated with *B. megaterium* (strain RAB2), which showed no significant difference. Nevertheless, the bacterial inoculation strategy may have been associated with signaling‐related responses, contributing to their stress tolerance (Li et al. 2022). Conversely, plants under better hydration were more exposed to solar radiation and, consequently, higher transpiration due to their increased growth and leaf production, potentially triggering CAT and APX activity to maintain redox homeostasis.

Water limitation can influence EO synthesis, production, and yield in medicinal plants (Li et al. 2020). However, studies have indicated that bacterial inoculation enhances EO production and chemical quality by stimulating an increase in antioxidant potential (Cappellari et al. 2020; Mirzaei et al. 2020; Semenzato and Fani 2024). An increase in EO quantity may favor the accumulation of compounds from primary and secondary metabolism that play significant roles in defense against adverse conditions, such as water stress (Asghari et al. 2020). Based on this, our third hypothesis proposed that the interaction between water stress and bacterial inoculation would significantly impact the production and chemical composition of *L*. *origanoides* EO, increasing the yield and/or concentration of key compounds linked to the physiological response of the plant to stress. Although quantitative EO production did not show significant differences among treatments, the EO chemical profile varied according to water availability and bacterial inoculation, partially supporting this hypothesis.

The quality of *L*. *origanoides* EO is predominantly characterized by terpenoids, a feature previously reported for the *Lippia* genus (Melo et al. 2022). These compounds are mainly derived from two biosynthetic pathways, the MEP and MVA pathways (Semenzato and Fani 2024), which can be stimulated in aromatic plants co‐cultivated with bacterial inoculants under abiotic stress (Mishra et al. 2018). In this study, inoculation with *Bacillus* strains was associated with changes in terpenoid production in *L*. *origanoides*, regardless of the water availability. The presence of exclusive compounds in inoculated plants suggests shifts in metabolic responses, potentially involving terpenoid‐related pathways (Gupta et al. 2021). This was reflected in the increase in the diversity of monoterpenes and sesquiterpenes, with variations depending on the bacterial species used for inoculation and the irrigation conditions (Hazrati et al. 2024).

Under water stress, plants treated with *B. megaterium* (RAB2) and the bacterial consortium showed higher contents of monoterpenes, such as thymol and carvacrol, and reduced contents of sesquiterpenes, including guaiol, β‐caryophyllene, and δ‐cadinene. This pattern likely reflects a reallocation of metabolic resources toward monoterpene biosynthesis, contributing to osmotic protection under stress conditions (Ghojavand et al. 2024). In contrast, under well‐watered conditions, the same treatments showed higher contents of sesquiterpenes, particularly β‐caryophyllene and germacrene D, which may contribute to the larvicidal potential of the EO, as both compounds have insecticidal activity against dengue and malaria vectors (Oliveira et al. 2022).

Terpene expression generally increases under water stress, contributing to antioxidant protection against ROS accumulation (Khakdan et al. 2021). In this study, inoculation under stress was associated with higher levels of defense‐related compounds, such as 1,8‐cineole and δ‐cadinene, which have been linked to antioxidant adaptation (Ancuceanu et al. 2024; Cai et al. 2021). β‐caryophyllene increased up to 10‐fold under stress compared to that under well‐watered conditions, suggesting a link among inoculation, antioxidant protection, and insecticidal potential (Oliveira et al. 2022).

Under well‐watered conditions, plants inoculated with *B. subtilis* (IN‐937b) showed a 114% higher thymol content than those under water stress. This increase was associated with greater leaf development and higher exposure to solar radiation, which may have stimulated antioxidant defenses, including CAT and APX activity. As thymol biosynthesis is closely associated with oxidative metabolism (Sun et al. 2024), this response may reflect the activation of antioxidant pathways. Considering the pronounced insecticidal activity of thymol, which disrupts metabolic processes and induces paralysis and death in target organisms (Aljameeli et al. 2025), the higher thymol content under this treatment suggests potential biotechnological applications.

Plants inoculated with *B. cereus* (C240) exhibited enhanced antioxidant capacity, together with indications of increased insecticidal and medicinal potential. Monoterpenoid antioxidants predominated across both water regimes. Under stress, compounds, such as terpinen‐4‐ol, carvacrol, α‐ and β‐copaene, α‐thujaplicinol, and butylated hydroxyanisole, may have been enhanced, reflecting stress‐related metabolic adjustments induced by inoculation (Cappellari et al. 2020). Although antioxidant defenses were prioritized, other bioactive compounds, including linalool, and insecticidal terpenes, such as caryophyllene oxide and bicyclogermacrene, were only detected in inoculated plants. β‐caryophyllene increased by approximately 20% under stress and more than tenfold under well‐watered conditions compared to non‐inoculated plants, indicating a potential association between inoculation and secondary metabolism (Semenzato and Fani 2024).

## 5 Conclusion

Water availability is a key factor determining the growth and physiological performance of *L*. *origanoides* under water‐deficit conditions. Bacterial inoculation contributed to plant adjustment under stress by enhancing stomatal conductance, increasing the leaf protein content, and promoting amino acid accumulation in the roots, reflecting metabolic reallocation, which also influenced the leaf‐to‐flower ratio. These changes improved the plant's tolerance to oxidative damage and overall resilience. Although EOY did not change, inoculation with *Bacillus* spp. modified its composition, leading to the formation of terpenoids with antioxidant and insecticidal activities. Further studies are needed to identify the mechanisms involved and enhance the potential applications of this interaction in therapeutic and industrial uses.

## Author Contributions

Conceptualization: H.C.A.O., E.B.S., L.P.N. and C.U. Methodology: H.C.A.O., E.B.S., and C.U. Investigation: H.C.A.O., L.B.S.P. and M.N.S. Resources: C.A.G.C., I.A.M.P. and E.B.S. Software: H.C.A.O. and L.B.S.P. Formal analysis: H.C.A.O., M.N., C.U., and M.M.M. Writing, review, and editing: H.C.A.O., L.B.S.P., E.B.S. and L.P.N. Visualization: F.C.L.S. Supervision: C.U. All authors critically reviewed the drafts and approved the final version for publication.

## Conflicts of Interest

The authors declare no conflicts of interest.

## Supporting information


**Table S1:** Analysis of variance of the effects of bacterial inoculation under different water availability treatments (VC%) in *Lippia origanoides* Kunth. The evaluated parameters include leaf 16 production and relative growth rates, compared using Tukey test (*p* ≤ 0.05).
**Table S2:** Analysis of variance of the effects of bacterial inoculation under different water availability treatments (VC%) in *Lippia origanoides* Kunth. The parameters evaluated include stomatal 27 conductance and SPAD index, compared by the Tukey test or determined based on the ranking (ARTool) compared by the Bonferroni method (*p* ≤ 0.05).
**Table S3:** Analysis of variance of the effects of bacterial inoculation under different water availability treatments (VC%) in *Lippia origanoides* Kunth. The evaluated parameters include the 30 levels of chlorophyll a, chlorophyll b, total chlorophyll, the chlorophyll a/b ratio and carotenoids, compared using Tukey test (*p* ≤ 0.05).
**Table S4:** Analysis of variance of the effects of bacterial inoculation under different water availability treatments (VC%) in *Lippia origanoides* Kunth. The evaluated parameters include the 33 levels of total soluble proteins in leaves, total soluble carbohydrates in leaves and roots, total free amino acids in leaves and roots and proline in leaves and roots, compared using Tukey test (*p* 34 ≤ 0.05).
**Table S5:** Analysis of variance of the effects of bacterial inoculation under different water availability treatments (VC%) in *Lippia origanoides* Kunth. The evaluated parameters include the 37 levels of Malondialdehyde (MDA) in leaves and roots and Hydrogen peroxide (H_2_O_2_) in leaves and roots, compared using Tukey test (*p* ≤ 0.05).
**Table S6:** Analysis of variance of the effects of bacterial inoculation under different water availability treatments (VC%) in *Lippia origanoides* Kunth. The evaluated parameters include the 40 levels of superoxide dismutase, catalase and ascorbato peroxidase in leaves, compared using Tukey test or Bonferroni adjustment (*p* ≤ 0.05).
**Table S7:** Analysis of variance of the effects of bacterial inoculation under different water availability treatments (VC%) in *Lippia origanoides* Kunth. The parameter evaluated is the production 43 of essential oil extracted from the leaves, compared using Tukey test (*p* ≤ 0.05).
**Table S8:** Chemical compounds of the essential oil extracted from *Lippia origanoides* Kunth subjected to co‐cultivation with bacterial inoculants of the Bacillus genus: Control (Non‐inoculated), 47 *B. cereus* (C240), *B. megaterium* (RAB2), *B. subtilis* (IN‐937b), and a consortium of bacterial inoculants under different water availability treatments: 75% (Hydrated) and 25% (Stress) at 37 48 days of co‐cultivation.
**Table S9:** Mean (*n* = 4) ± standard error of untransformed biological values for stomatal conductance (g_s_) at Time 4.
**Table S10:** Mean (*n* = 4) ± standard error of untransformed biological values for Superoxide dismutase (SOD).

## Data Availability

All data supporting the findings of this study are included in the article and in the Supporting Information file. Additional data will be made available from the corresponding author upon reasonable request.
