# Supplementary material for: Influence of Water Availability and Bacillus Inoculation on Morphophysiology, Biochemistry and Secondary Metabolite in Lippia origanoides Kunth
Source: Physiol Plant. 2026 Sep 24;178(5):e71132. doi: 10.1111/ppl.71132 (PMC13612156; doi:10.1111/ppl.71132)
Supplement: Supplementary file 1 [file PPL-178-e71132-s001.pdf]

Supplementary Data

Influence of water availability and *Bacillus* inoculation on morphophysiology, biochemistry and secondary metabolite in *Lippia origanoides* Kunth

Henarmmany Cristina Alves de Oliveira [a], Larisse Bianca Soares Pereira [e], Manuela Neri [a], Igor Alexsander de Melo Pimentel [b], Claudio Augusto Gomes da Câmara [c], Marcilio Martins de Moraes [c], Flávia Carolina Lins da Silva [a], Elineide Barbosa de Souza [a], Luiz Palhares Neto [d] *, Cláudia Ulisses[a] *

[a] Department of Biology, Federal Rural University of Pernambuco (UFRPE), Recife, Pernambuco, Brazil.
[b] Department of Agronomy, Federal Rural University of Pernambuco (UFRPE), Recife, Pernambuco, Brazil.
[c] Department of Chemistry, Federal Rural University of Pernambuco (UFRPE), Recife, Pernambuco, Brazil.
[d] Department of Biological Sciences, Southwest Bahia State University (UESB), Jequié, Bahia, Brazil.
[e] National Institute of Biotechnology for the Mineral Sector (INABIM), Recife, Pernambuco, Brazil.

* Correspondence
Luiz Palhares Neto

E-mail: luiz.neto@uesb.edu.br

Cláudia Ulisses

E-mail: claudia.ucsilva@ufrpe.br

Table S1. Analysis of variance of the effects of bacterial inoculation under different water availability treatments (VC%) in *Lippia origanoides* Kunth. The evaluated parameters include leaf production and relative growth rates, compared using Tukey test ($p \leq 0.05$).

Fixed effects		*Time interval 1*		*Time interval 2*		*Time interval 3*		*End of the experiment*
	DF	Leaf production	Relative growth rate	Leaf production	Relative growth rate	Leaf production	Relative growth rate	Leaves/ flowers
Water availability	1	9.86^{e-06}***	**0.00680****	**<2^{e-16} *****	**4.38^{e-13}*****	**1.3^{e-07}*****	<2^{e-16} ***	**1.21^{e-06} *****

Bacterial inoculation	4	0.00274**	**0.00875****	0.184	0.247	0.942	0.2613	0.210
Water availability × Bacterial inoculation	4	**0.00060*****	0.67287	0.243	0.265	0.305	**0.0472***	0.144

Degrees of freedom: DF; Significance: (***) p<0.001; (**) p<0.01 and (*) p< 0.05 via ANOVA.

Table S2. Analysis of variance of the effects of bacterial inoculation under different water availability treatments (VC%) in *Lippia origanoides* Kunth. The parameters evaluated include stomatal conductance and SPAD index, compared by the Tukey test or determined based on the ranking (ARTool) compared by the Bonferroni method ($p \leq 0.05$).

Fixed effects		*time 1*	*time 2*	*time 3*	*time 4*	*time 1*	*time 2*	*time 3*	*time 4*
	DF	Stomatal conductance				SPAD Index			
Water availability	1		**2.3065^{e-6} *** **	0.34796	0.00101**		**1.6074^{e-7}*****	**1.2701^{e-10}*****	0.209882
Bacterial inoculation	4	0.2375706	0.14872	0.68806	0. 43388	0.6693733	0.18748	0.87973	0.767785
Water availability × Bacterial inoculation	4		0.58314	0.50462	**0.02619***		0.54299	0.58966	**0.029242***

Degrees of freedom: DF; Significance: (***) p<0.001; (**) p<0.01 and (*) p< 0.05 via ANOVA.

Table S3. Analysis of variance of the effects of bacterial inoculation under different water availability treatments (VC%) in *Lippia origanoides* Kunth. The evaluated parameters include the levels of chlorophyll *a*, chlorophyll *b*, total chlorophyll, the chlorophyll *a/b* ratio and carotenoids, compared using Tukey test ($p \leq 0.05$).

Fixed effects	DF	Chlorophyll *a*	Chlorophyll *b*	Chlorophyll total	Chlorophyll *a/b*	Carotenoids
Water availability	1	0.05646	0.51519	0.09241	**0.01761***	0.17442
Bacterial inoculation	4	0.41536	0.80196	0.51266	0.43046	0.52863

Water availability × Bacterial inoculation	4	0.44960	0.38604	0.41094	0.56464	0.60294

Degrees of freedom: DF; Significance: (***) p<0.001; (**) p<0.01 and (*) p< 0.05 via ANOVA.

Table S4. Analysis of variance of the effects of bacterial inoculation under different water availability treatments (VC%) in *Lippia origanoides* Kunth. The evaluated parameters include the levels of total soluble proteins in leaves, total soluble carbohydrates in leaves and roots, total free amino acids in leaves and roots and proline in leaves and roots, compared using Tukey test ($p \leq 0.05$).

Fixed effects	DF	Total soluble proteins in leaves	Total soluble carbohydrates in leaves	Total soluble carbohydrates in roots	Total free amino acids in leaves	Total free amino acids in roots	Proline in leaves	Proline in roots
Water availability	1	**8.17^{e-08} *****	0.353	**1.73^{e-4} *****	**0.0134***	2.12^{e-06}***	**7.67^{e-5} *****	**2.38^{e-7} *****
Bacterial inoculation	4	**0.00127 ****	0.775	0.254828	0.8778	0.69371	0.522	0.861
Water availability × Bacterial inoculation	4	0.30741	0.919	0.108741	0.5302	**2.28^{e-3}****	0.557	0.140

Degrees of freedom: DF; Significance: (***) p<0.001; (**) p<0.01 and (*) p< 0.05 via ANOVA.

Table S5. Analysis of variance of the effects of bacterial inoculation under different water availability treatments (VC%) in *Lippia origanoides* Kunth. The evaluated parameters include the levels of Malondialdehyde (MDA) in leaves and roots and Hydrogen peroxide (H_2O_2) in leaves and roots, compared using Tukey test ($p \leq 0.05$).

Fixed effects	DF	Malondialdehyde in leaves	Malondialdehyde in roots	Hydrogen peroxide in leaves	Hydrogen peroxide in roots
Water availability	1	**0.0397***	0.059	**2.14^{e-4}*****	**1.4^{e-4}*****
Bacterial inoculation	4	0.3308	0.108	0.637167	0.46000
Water availability × Bacterial inoculation	4	0.2327	0.414	0.286214	0.14526

Degrees of freedom: DF; Significance: (***) p<0.001; (**) p<0.01 and (*) p< 0.05 via ANOVA.

Table S6. Analysis of variance of the effects of bacterial inoculation under different water availability treatments (VC%) in *Lippia origanoides* Kunth. The evaluated parameters include the levels of superoxide dismutase, catalase and ascorbato peroxidase in leaves, compared using Tukey test or Bonferroni adjustment ($p \leq 0.05$).

Fixed effects	DF	Superoxide dismutase	Catalase	Ascorbato peroxidase
Water availability (VC%)	1	1.0965^{e-10} ***	2.54^{e-13} ***	4.00^{e-10}***
Bacterial inoculation	4	4.0460^{e-6} ***	8.37^{e-7} ***	1.44^{e-5}***
Water availability × Bacterial inoculation	4	**<2.22^{e-16} *** **	**6.68^{e-7} *** **	**7.52^{e-6}*** **

Degrees of freedom: DF; Significance: (***) p<0.001; (**) p<0.01 and (*) p< 0.05 via ANOVA.

Table S7. Analysis of variance of the effects of bacterial inoculation under different water availability treatments (VC%) in *Lippia origanoides* Kunth. The parameter evaluated is the production of essential oil extracted from the leaves, compared using Tukey test ($p \leq 0.05$).

Fixed effects	DF	Essential oil production
Water availability	1	0.2748
Bacterial inoculation	4	0.2049
Water availability × Bacterial inoculation	4	0.0688

Degrees of freedom: DF; Significance: (***) p<0.001; (**) p<0.01 and (*) p< 0.05 via ANOVA.

Table S8. Chemical compounds of the essential oil extracted from *Lippia origanoides* Kunth subjected to co-cultivation with bacterial inoculants of the *Bacillus* genus: Control (Non-inoculated), *B. cereus* (C240), *B. megaterium* (RAB2), *B. subtilis* (IN-937b), and a consortium of bacterial inoculants under different water availability treatments: 75% (Hydrated) and 25% (Stress) at 37 days of co-cultivation.

Chemical components of essential oil (EO)	RI[a]	RI[b]	Inoculation with bacteria of the genus *Bacillus* under different water availability treatments									
			Non-inoculated		*B. cereus* (C240)		*B. megaterium* (RAB2)		*B. subtilis* (IN-937b)		Consortium	
			Hydrated	Stress	Hydrated	Stress	Hydrated	Stress	Hydrated	Stress	Hydrated	Stress
α-Thujene	925	930	0.16±0.01		0.11±0.01		0.12±0.01		0.51±0.03		0.38±0.01	
α-pinene	930	932	0.09±0.004		0.13±0.007		0.18±0.01		0.32±0.01		0.18±0.02	
Myrcene	985	988	0.8±0.01		0.47±0.02	0.38±0.03	0.33±0.01		1.13±0.02		0.33±0.01	
αTerpinene	1016	1014	0.52±0.01		0.91±0.04		0.75±0.03	0.33±0.01	1.08±0.04		0.52±0.02	
o-cymene	1020	1022	5.3±0.14	1.01±0.01	3.15±0.01	1.45±0.05	2.5±0.11		7.36±0.14	6.17±3.36	3.35±0.04	
1,8-Cineole	1022	1026		0.55±0.02	0.78±0.03	0.36±0.01	0.24±0.03	1.8±0.01		0.98±0.01	0.53±0.09	
β-(E)-Ocimene	1040	1044	0.43±0.01						0.69±0.02			
γ-terpinene	1051	1054	5.79±0.05	1.95±0.01	7.63±0.18	2.03±0.03	2.6±0.04	1.56±0.02	7.13±0.06	7.15±2.50	4.57±0.05	
cis-sabinene hydrate	1063	1065			0.24±0.01				0.26±0.02			0.43±0.01
Linalool	1098	1095			0.33±0.01	0.7±0.03	0.41±0.004	0.3±0.02			0.31±0.01	
Terpinen-4-ol	1177	1174		0.6±0.03	0.42±0.01	0.63±0.03	0.64±0.01	0.52±0.002	0.33±0.01		0.86±0.01	
Methyl thymol ether	1231	1232		4.34±0.03	5.2±0.04	4.48±0.05	3.12±0.03	3.55±0.02		4.4±1.05	4.09±0.01	3.37±0.06
Methyl carvacryl ether	1242	1241			0.68±0.01	0.43±0.02	0.37±0.01	0.46±0.01	0.41±0.006		0.44±0.002	
Thymol	1291	1289	12.2±0.26	7.32±0.14	5.64±0.11	5.71±0.04	7.22±0.06	7.57±0.03	12.17±0.04	5.67±0.10	6.56±0.06	7.49±0.12
Carvacrol	1297	1298	69.51±0.69	66.86±0.64	52.73±0.41	56.72±0.19	66.66±0.53	72.96±0.29	64.78±0.16	60.32±2.73	61.6±0.22	78.58±0.3
α-copaene	1374	1374			0.55±0.02	0.82±0.04	0.54±0.01		0.6±0.02		0.91±0.01	
α-gurjunene	1410	1409			0.27±0.27							
β-copaene	1432	1430		1.19±0.11	0.65±0.01	1.03±0.03	1.2±0.03	0.58±0.04			0.72±0.03	0.8±0.03
Allo-aromadendrene	1458	1458			0.29±0.01	0.14±0.01						
α-Humulene	1477	1479			0.3±0.01	0.36±0.01						
γ-Muurolene	1605	1600		1.4±0.03		0.72±0.02	0.46±0.02				0.36±0.01	
Guaiol	1644	1640		3.25±0.09	4.41±0.02	4.84±0.03	1.75±0.02	1.09±0.02		3.2±0.80	3.05±0.04	0.52±0.01
Epi-α-Muurolol	1432	1430				0.52±0.01				0.47±0.01		
β-caryophyllene	1413	1417	0.53±0.02	5.8±0.07	6.17±0.04	7.03±0.06	4.7±0.06	3.8±0.04	0.51±0.06	5.43±1.53	5.29±0.07	4.96±0.05
Germacrene D	1451	1452		1.41±0.04	5.36±0.12	2.71±0.03	0.91±0.04			2.09±0.60	1.54±0.05	0.14±0.02
Bicyclogermacrene	1485	1484			0.84±0.02							
β-Bisabolene	1501	1500	0.55±0.02			0.65±0.03			0.44±0.05			
γ-Cadinene	1507	1509				0.38±0.02						
δ-Cadinene	1516	1513		1.72±0.03	0.73±0.02	2.02±0.01	1.15±0.03	0.32±0.004		1.67±0.46	1.25±0.05	0.54±0.02
Caryophyllene oxide	1527	1522				0.35±0.01	0.7±0.03	0.78±0.02			0.45±0.02	
α-Thujaplicinol	1590	1582		1.32±0.04	1.1±0.02	3.27±0.01	2.26±0.05	2.18±0.01			1.43±0.04	2.12±0.03

Butylated hydroxy anisole	1505	1505	2.73±0.05		0.46±0.02	1.05±0.05	0.95±0.03	0.95±0.01	2.18±0.04		0.69±0.01	0.71±0.01
Total monoterpenes			94.81±0.83	82.65±0.44	78.40±0.35	72.88±0.25	85.12±0.31	89.05±0.22	96.16±0.03	85.90±3.26	83.73±0.25	89.89±0.21
Total sesquitepenes			3.80±0.04	16.08±0.31	21.11±0.16	25.87±0.28	14.61±0.17	9.69±0.14	3.73±0.06	13.16±3.10	15.67±0.22	9.75±0.05
Total identification (%)			98.61	98.73	99.51	98.74	99.73	98.74	99.89	99.05	99.41	99.64

Retention index (RI): a – Calculated from retention times relative to a homologous series of n-alkanes on a 30 m DB-5 column; b – Values from the literature. Compound identification was based on retention times, mass spectral comparison with databases (NIST 14, WILEY 14th), and authentic standards (monoterpenes: α-pinene, myrcene, α-terpinene, 1,8-cineole, linalool, thymol, carvacrol; sesquiterpenes: β-caryophyllene, Allo-aromadendrene, α-humulene, germacrene D, bicyclogermacrene, and caryophyllene oxide).

Table S9. Mean (n = 4) ± standard error of untransformed biological values for stomatal conductance (g_s) at time 4.

Inoculation with bacteria of the genus *Bacillus*	Hydrated	Stress
Non-inoculated	0.269 ± 0.006	0.207 ± 0.007
B. cereus (C240)	0.238 ± 0.012	0.205 ± 0.005
B. megaterium (RAB2)	0.225 ± 0.006	0.217 ± 0.022
B. subtilis (IN-937b)	0.237 ± 0.023	0.244 ± 0.004
Consortium	0.263 ± 0.010	0.163 ± 0.038

Table S10. Mean (n = 4) ± standard error of untransformed biological values for Superoxide dismutase (SOD).

Inoculation with bacteria of the genus *Bacillus*	Hydrated	Stress
Non-inoculated	128150.03 ± 5327.66	7055.05 ± 3162.44
B. cereus (C240)	22408.68 ± 5730.66	1477.69 ± 104.85
B. megaterium (RAB2)	8711.61 ± 4375.47	948.52 ± 84.52
B. subtilis (IN-937b)	17742.00 ± 5519.92	1889.09 ± 966.18
Consortium	12408.42 ± 3503.61	3529.90 ± 105.93
